# A semi-automated motion-tracking analysis of locomotion speed in the *C. elegans* transgenics overexpressing beta-amyloid in neurons

**DOI:** 10.3389/fgene.2014.00202

**Published:** 2014-07-04

**Authors:** Kevin Machino, Christopher D. Link, Susan Wang, Hana Murakami, Shin Murakami

**Affiliations:** ^1^Department of Basic Sciences, College of Osteopathic Medicine, Touro University CaliforniaVallejo, CA, USA; ^2^Institute of Behavioral Genetics, University of Colorado, BoulderBoulder, CO, USA

**Keywords:** Alzheimer's disease, beta amyloid, age-related memory impairment (AMI), frailty, behavioral aging, motion tracking, automated system, Alzheimer's disease in children

## Abstract

Multi-Worm Tracker (MWT) is a real-time computer vision system that can simultaneously quantify motional patterns of multiple worms. MWT provides several behavioral parameters, including analysis of accurate real-time locomotion speed in the nematode, *Caenorhabditis elegans*. Here, we determined locomotion speed of the Alzheimer's disease (AD) transgenic strain that over-expresses human beta-amyloid1-42 (Aβ) in the neurons. The MWT analysis showed that the AD strain logged a slower average speed than the wild type (WT) worms. The results may be consistent with the observation that the AD patients with dementia tend to show deficits in physical activities, including frequent falls. The AD strain showed reduced ability of the eggs to hatch and slowed hatching of the eggs. Thus, over-expression of Aβ in neurons causes negative effects on locomotion and hatchability. This study sheds light on new examples of detrimental effects that Aβ deposits can exhibit using *C. elegans* as a model system. The information gathered from this study indicates that the motion tracking analysis is a cost-effective, efficient way to assess the deficits of Aβ over-expression in the *C. elegans* system.

## Introduction

Alzheimer's disease (AD) is a type of amyloidosis and is the major neurodegenerative disorder that causes dementia. Amyloidosis is characterized by fibril deposits that contain at least 30 fibril proteins in humans and 10 fibril proteins in animals, according to the 2012 the Nomenclature Committee of the International Society of Amyloidosis (Sipe et al., 2012). Of them, accumulation of Aβ has been observed in the AD and congophilic cerebral angiopathy (CAA) (Selkoe, 2001; Revesz et al., 2003). AD leads to cognitive deficits due to abnormal deposits of β-amyloid (Aβ) peptides in the brain, while CAA is characterized by vascular deposits in the central nervous system, in which hemorrhage is a major clinical feature (Samarasekera et al., 2012). CAA is often observed in AD patients, in which case it has been diagnosed as AD (Wright, 2013).

In AD, deposits of the Aβ peptides arise from the proteolytic processing of amyloid precursor proteins (APP), commonly observed in patients with AD (Nicholson et al., 2012). Aβ deposits and tau tangles are well known hallmarks for AD, which may trigger inflammation worsening the disease (Nicholson et al., 2012; Jack and Holtzman, 2013). Aβ toxicity may be linked to tau hyperphysphorylation observed in tau tangles (Lloret et al., 2011; Ermak and Davies, 2013). Although it is believed that Aβ is involved in initiation of AD in the presence of tau pathologies and that tau is correlated with severity of AD (reviewed in LaFerla, 2010; Castillo-Carranza et al., 2014), molecular mechanisms of AD remain unclear. Details of clinical pathogenesis and biomarkers have been reviewed elsewhere (Jack and Holtzman, 2013).

This study aimed to assess the effects of Aβ toxicity on a behavioral parameter, average speed of movement. A transgenic strain overexpressing the signal peptide::Aβ^1−42^ in the neurons (Dosanjh et al., 2010; Lublin and Link, 2013) was used for this study. Over-expression of Aβ in the neurons may affect the serotonin system (Dosanjh et al., 2010). Previously, locomotion has been assessed in a manual assay that measures the rate of body bend, which is an indirect measure with relatively high variability (Dosanjh et al., 2010; Lublin and Link, 2013); the manual assay may have been missed early signs of motility defect. In addition, another neural Aβ strain is tagged by a behavioral marker (i.e., roller phenotype) (McColl et al., 2012), which is difficult to assess locomotion speed. We used behavioral tracking software, Multi Worm Tracker (MWT) (Swierczek et al., 2010), to record and collect data on the speed. MWT is a program designed to quantify the behavior of multiple worms on a petri plate with minimal human effort. The main advantage is that it allows for a more accurate and detailed assessment of locomotion speed compared to the traditional method that measures body bends. Thus, our hypothesis is that the strain overexpressing Aβ in the neurons show defects in locomotion speed. We reason that motion tracking analysis, including MWT analysis, should contribute to understanding the harmful effects of Aβ toxicity in *C. elegans*.

## Materials and methods

### Strains, media, and staining

Wild-type strain, N2, was used (referred to as WT strain). As a control, we also used the *smg-1*^*ts*^ strain [*smg-1*^*ts*^*(cc546)*], which generated similar results as N2 in the assays (i.e., locomotion and hatchability) used in this study (data not shown). The strain CL2355 [*smg-1*^*ts*^*(cc546)*; *snb-1::Aβ*_1−42_::long 3′-UTR] that utilizes the *C. elegans* promotor of the synaptobrevin (*snb-1*) gene to cause a pan-neuronal overexpression of the signal peptide::Aβ^1−42^ (Dosanjh et al., 2010; Lublin and Link, 2013) was used (referred to as AD strain). For simplicity, the transgenic strain CL2355 is referred to as the AD strain for the duration of the paper. All strains were maintained at 15°C in a nematode growth media (NGM) spotted with *Escherichia coli*, OP50, as a food source (Murakami et al., 2005). The following procedure adopted from Stiernagle (2006) was used to prepare NGM plates. Before autoclaving, 17 g agar, 2.5 g peptone, 3 g NaCl, and 975 mL distilled H_2_O was added to a flask and covered with aluminum foil. Then the flask was autoclaved for 50 min. After autoclaving the mixture, 1 mL of 1 M CaCl_2_, 1 mL of 5 mg/mL cholesterol in ethanol, 1 mL of 1 M MgSO_4_, and 25 mL of 1 M KPO_4_ (pH 6.0) was added to the flask. Then petri plates were filled 2/3 with agar. Plates were left at room temperature for 2–3 days to allow excess moisture to evaporate. For immunofluorescence staining, we used the procedure described in Link (1995). Transgenic worms were fixed, permeabilized, and stained with the anti-Aβ monoclonal antibody 4G8 and anti-TOR-2 polyclonal sera as a counterstain. DNA was visualized using 4′,6-diamidino-2-phenylindole (DAPI).

### Growth conditions

Strains were grown on NGM agar plates at 15°C. To assess hatchability, eggs were layed on NGM plates at 15°C and incubated at the temperature indicated in the text (15 or 25°C). Unhatched eggs were counted after 24 h or the time specified in the text. Hatched eggs (larvae) were also counted to confirm the result. To prepare adult worms, eggs were layed and grown into adults for 4 days. Adults at the age of day 5 (i.e., 1 day after they start to lay eggs) was defined as “younger adults.” Adults at the age of day 7 was defined as “middle-aged adults.” Adults at the age of day 13–14 was defined as “older adults.”

### Multi worm tracker (MWT) analysis

Videos of the worms were recorded under a stereomicroscope using ToupView, a video capturing software (Amscope.com, Irvine, CA). The parameters of 3 min for duration and 6 s for bin size was set when capturing the video, which created a video file that was 35 s in length. To analyze the speed of the worms, the video file was uploaded into MWT, a behavioral tracking software (Swierczek et al., 2010). In a typical assay, about five worms in a microscopic field was computed and average speed of the worms were calculated. A measure for speed (in pixels/s) was made at a series of ages indicated in the text. We used the conversion rate in the system: 1 pixel/s = 0.035 cm/s at the images of 72 PPI (pixels per inch). Death was determined by observing no movement of the worms. Statistical analysis has been performed by ANOVA using NCSS 2007 statistics software (NCSS, LLC, Kaysville, Utah, USA).

## Results

**Figure 1:**
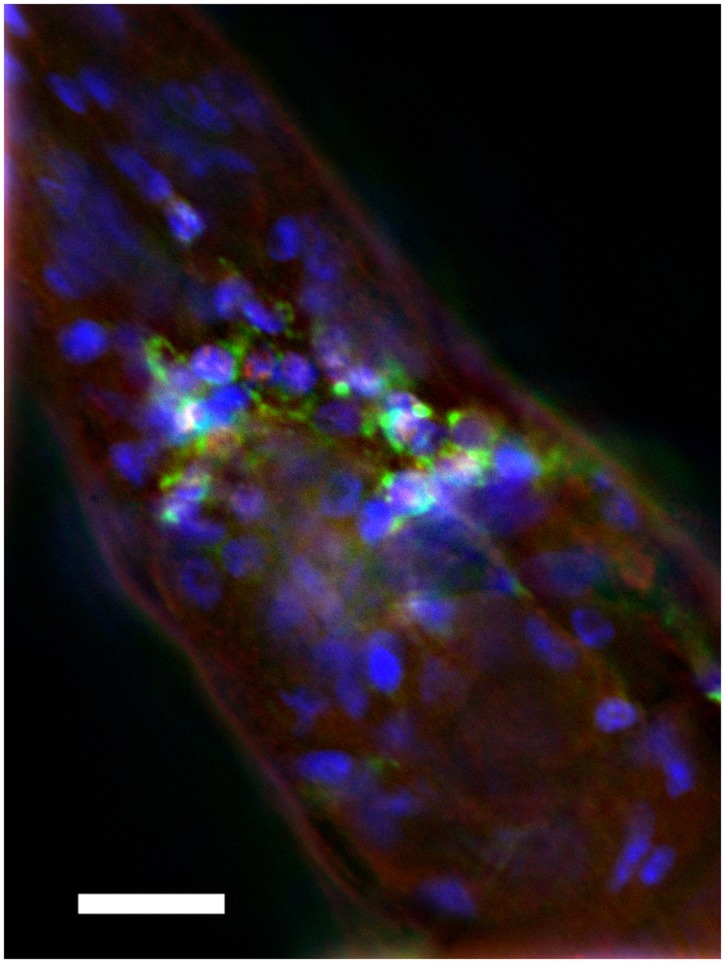
**Intraneuronal deposition of Aβ in transgenic *C. elegans***. Anterior region of transgenic *C. elegans* is shown. The strain was immune-stained by anti-Aβ monoclonal antibody 4G8 (green), anti-TOR-2 polyclonal sera (red), and DAPI (blue). Note cytoplasmic accumulation of Aβ in nerve ring neurons. Size bar = 10 μM.

We sought to assay the locomotion of the transgenic *C. elegans* strain CL2355 (Dosanjh et al., 2010), which expresses a human Aβ_42_ minigene under the control of the pan-neuronal synaptobrevin (*snb-1*) promoter. Expression of Aβ using this promoter leads to intraneuronal deposition of Aβ, particularly detectable in the nerve ring area (Figure 1).

Video images of the worms on NGM agar were captured for 3 min and the speed of the worms was determined using MWT. We compared the results of the WT (wild type) and AD strains (over-expressing amyloid beta) (Materials and Methods). As described in the Method, adults that had grown for 5 days were defined as “younger adults.” Adults that had grown for 7 days were defined as “middle-aged adults.” Adults that had grown for 13 days were defined as “older adults.”

### The AD transgenic strain showing slower movement

**Figure 2:**
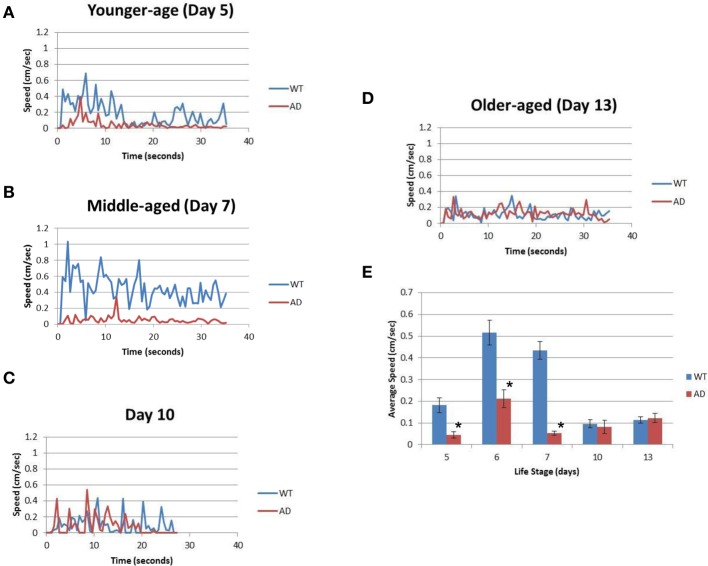
**The AD strain exhibits slower speeds than WT in the life cycle. (A)** The graph represents the locomotion speed of adult worms measured at the age of day 5 (younger age). **(B)** The graph represents the speed for worms measured at the age of day 7 (middle age). **(C)** The graph represents the speed for middle-aged worms measured at the age of day 10. **(D)** The graph represents the speed for older adult worms measured at the age of day 13 (older age). **(E)** Comparison of the average speeds for WT (blue) and AD (red) worms at various ages over the course of their life span. Error bars indicate standard error of the mean. ^*^p > 0.0001. WT worms (blue); and AD worms (red). See also Table 1.

Table 1 summarizes the results. For the younger adults (day 5), WT had a mean speed of 0.18 ± 0.15 cm/s whereas the AD had a mean speed of 0.05 ± 0.06 cm/s (*p* > 0.0001; Figure 2A; Table 1). Slow locomotion speed in the AD strain compared to WT was observed from day 5 (younger adults) to day 7 (middle age) (*p* > 0.0001; Figures 2A,B; Table 1). Interestingly, there was a peak of locomotion speed at the age of day 5 (Figure 2E), which was consistent with previous study as assessed in a classical assay that measured body bends, an indicator of locomotion speed (Murakami et al., 2008).

**Table 1:** Locomotion speed (mean ± standard deviation) as assessed by MWT analysis for various stages in the life cycle (*p < 0.0001).

**Life stage (day #)**	**Type of strain**	**Average speed (cm/s)**
Younger adult (day 5)	WT	0.18 ± 0.15
	AD	0.05 ± 0.06^*^
(day 6)	WT	0.52 ± 0.25
	AD	0.21 ± 0.17^*^
Middle-aged (day 7)	WT	0.43 ± 0.19
	AD	0.05 ± 0.05^*^
(day 10)	WT	0.10 ± 0.11
	AD	0.08 ± 0.12
Older adult (day 13)	WT	0.11 ± 0.07
	AD	0.12 ± 0.07

**Figure 3:**
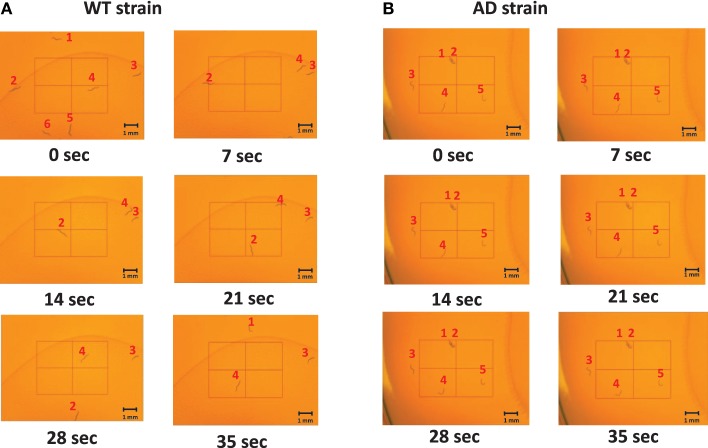
**Images of middle-aged (day 7) worms on the NGM plates at various time points**. The images captured were taken every 7 s from 0 to 35 s. The worms were labeled 1–6 for the WT and 1–5 for the AD strain so that the difference in movement could be seen between and amongst frames. **(A)** An example of the WT worms. They moved throughout the plate as time progressed. **(B)** An example of the AD worms. They hardly moved as time elapsed.

Slow locomotion speed in the AD strain was also evident in the still images (Figure 3) and Supplementary Video (Supplementary Figure). In the sill images taken every 7 s (Figure 2), the WT worms were located in different positions (Figure 3A), while the AD worms were nearly stagnant from 0 to 35 s (Figure 3B). Thus, a difference in the worm movements was clearly visible.

Older worms at day 10 and 13 showed similar results for the WT and AD strains. Day 10 WT worms had an average speed of 0.10 ± 0.11 cm/s whereas Day 10 AD worms moved at an average speed of 0.08 ± 0.12 cm/s for four worms (Figure 2C; Table 1). Finally, in the older adult at the age of Day 13, WT worms logged an average speed of 0.11 ± 0.07 cm/s and Day 13 AD worms showed similar locomotion speed (0.12 ± 0.07 cm/s) (Figure 2D; Table 1). Overall, worms with overexpressed Aβ resulted in slower movement up to middle-aged (Figure 2).

### Aβ toxicity in the embryonic stage

**Figure 4:**
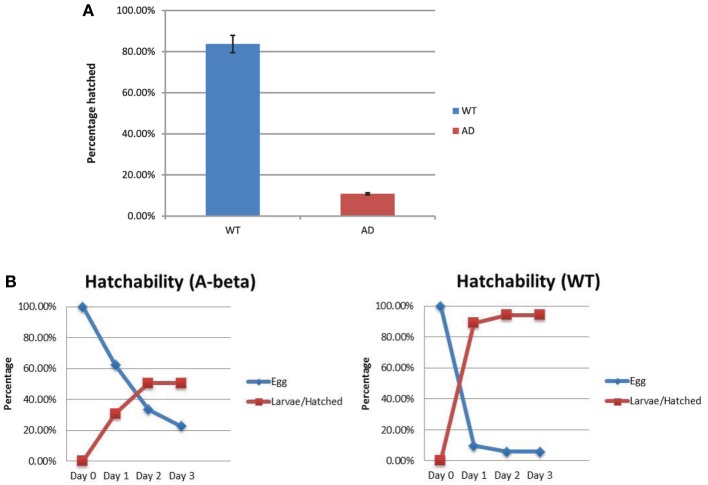
**Rate of egg hatching (hatchability) in the WT and AD strains. (A)** Percentage of eggs unhatched after 24 h. There were more AD eggs (red) that remained unhatched compared to the WT eggs (blue). **(B)** Time course of the percentage of eggs that hatched from day 0 (the day of egg lay) to day 3 for both strains. Error bars indicate standard error of the mean.

Hatching of the eggs from the WT (N2) and AD (CL2355) strains were compared. We measured total number of unhatched eggs 24 h at restrictive temperature (25°C) after egg lay. We also counted hatched larvae to confirm the results. In the WT strain, there were 16.3% (37/227) eggs that remained unhatched and 83.7% (190/227) hatched (Figure 4A). For the AD strain, 89.2% (182/204) eggs remained unhatched and 10.8% (22/204) hatched. The rate of hatching in the control strain (*smg-1*^*ts*^), was similar to WT (data now shown), excluding the possibility that the background mutation lowered the rate of hatching. Thus, the AD strain showed an approximately 7.8 folds lower hatching after 24 h of egg lay. We also investigated the time course of hatchability at permissive temperature, 15°C. Most of the eggs in the WT strain hatched 1 day after egg lay (Day 2; Figure 4B). In contrast, the AD strain hatched much slower than the WT strain, taking 2 days after egg lay (Day 3; Figure 4B). Thus, the AD strain shows reduced hatching and a delayed timing of egg hatching. We also observed a low rate of fecundity (Blood size: WT, 248 ± 22, *n* = 10; AD, 50 ± 12, *n* = 20; *p* < 0.001).

## Discussion

In this study, we used MWT analysis to measure locomotion speed. Compared to a manual assay to measure the number of body bends, MWT analysis provides a more accurate measure of multiple worms for a longer time (MWT for 3 min; Manual assay for 1 min). Young wild-type worm (locomotion speed: 0.18 ± 0.15 cm/s) was within the range of previously reported results (lower range of 0.13 cm/s, Ramot et al., 2008; upper range of 0.22 cm/s, Ryu and Samuel, 2002). They have also reported locomotion speed, ranging from 0.14 to 0.33 cm/s at the temperatures ranging from 17 to 27°C. The groups used other worm tracking systems, which suggests that MWT analysis results are consistent with those from the other worm tracking systems. It is worth noting that our MWT system is one of the most cost effective systems. The total cost was estimated to be up to US$200 for a routine USB-camera (as of April, 2014; our cost was zero since we recycled the camera, excluding the cost of software, computer, and microscope), which is much less than the cost estimated for MWT elsewhere (US$7,000) (Husson et al., 2012).

The AD strain showed an approximately 7.8-fold decrease in hatching compared to the WT strain. Likewise, the AD strain showed reduced hatching even at the permissive temperature, where Aβ is expressed, but at a lower level. The reduced level of hatching seen in the AD strain could be attributed to Aβ overexpression, suggesting the negative effect of Aβ on embryogenesis due to its toxicity. It is worth noting that some CL2355 worms are completely sterile (no eggs laid). This is a surprising observation if the defects in the CL2355 strain are restricted to neurons, as fertility in *C. elegans* is not strongly neuronally regulated. The fertility/sterility phenotypes of CL2355 were observed in the precursor extrachromosomal line, suggesting that these defects are not due to gene disruption caused by chromosomal integration of the transgene. We also observed that worms show larval arrest and death, suggesting defects in development caused by Aβ (data not shown).

The findings also suggest that Aβ accumulation has detrimental effects on locomotion speed in the adult life cycle, except for late-life in which all worms move poorly. In younger adults and middle-aged adults (corresponding to Day 5 and 7, respectively), the AD worms exhibited a slower speed than the WT worms (Figure 1). The middle-aged AD worms showed the greatest amount of difference from that of the WT worms; the approximate 8.3-fold difference in speed indicates that the presence of Aβ is correlated with the slower movement in the worms. This may be consistent with frailty seen in the Alzheimer's patients (Koch et al., 2013; Kulmala et al., 2014) and gait problems in the mouse models of AD (APP and APP/PS1 mice) (Lalonde et al., 2012; Wang et al., 2012a), though some other mice models [APP23, J20, APP + PDAPP, PS1 [Tg2576 + PS1 (M146L)], TgCRND8, TG2576, and 3 × Tg-AD mice] show increased locomotion due to aggression and other behavioral problems (Webster et al., 2014). Thus, locomotion is not always defective in AD patients and in AD models but rather altered. It is critical to assess locomotion in each AD system. In addition to our finding, locomotion defects have been observed in the fruit fly model of Aβ toxicity, while paralysis has been observed in the other strains over-expressing Aβ in the muscles and in the neurons (McColl et al., 2012; Wang et al., 2012b; Lublin and Link, 2013; Prüßing et al., 2013; Carrillo-Mora et al., 2014). Technical difficulties in the previous studies have been discussed above.

This study provides evidence that Aβ toxicity affects the embryonic stage as well as the adult phases. Since the AD transgenic strain used in this study has Aβ overexpression, the data can be used to assess the effects of Aβ toxicity on embryonic and behavioral parameters. Assessing the hatchability and speed of the worms allows for the analysis of the strains and the impact Aβ toxicity has on embryonic health and movement. The research will be beneficial as AD is one of the most common causes of dementia, and the number of Americans aged 65 and older affected by AD is predicted to triple by 2050 (Hebert et al., 2013). Currently, there are drugs there are drugs that can treat memory impairment in AD patients, but there are no cure for the disease itself. It is worth noting that a chaperone, HSP-16, is strongly associated with the Aβ deposits in the neuronal expression lines (reviewed in Lublin and Link, 2013). The future direction of this study aims to explore the relationship between AD drugs and potential Aβ clearance. The hope is that future studies will demonstrate the effectiveness of current FDA-approved AD treatment interventions to help alleviate the Aβ buildup seen in AD patients and enhance future treatments by potentially slowing or stopping the disease.

### Conflict of interest statement

The authors declare that the research was conducted in the absence of any commercial or financial relationships that could be construed as a potential conflict of interest.

## Supplementary material

Supplementary Video **Video images of day 7 worms shown in Figure 2. (Video 1)**. Video shows the WT strain (File name: Machino et al. WT strain Day 7.mwv). **(Video 2)** Video shows the AD strain (File name: Machino et al. AD strain Day 7.mwv). As described in Materials and Methods, 3 min-long videos were shortened into 35 s in length. Due to the conversion from AVI to MP4 format to reduce the size of the files, the video have lower quality compared to the originals.
